# Spinal cord compression by extramedullary hematopoiesis in beta‐thalassemia major

**DOI:** 10.1002/ccr3.2924

**Published:** 2020-07-28

**Authors:** Lobna Ben Ammar, Hanene Ferjani, Kaouther Maatallah, Sonia Bouallegue, Hend Riahi, Dhia Kaffel, Wafa Hamdi

**Affiliations:** ^1^ Department of Rheumatology Mohammed Kassab National Institute of Orthopaedics Mannouba Tunisia; ^2^ Faculty of Medicine of Tunis University Tunis El Manar Tunis Tunisia; ^3^ Departments of General Medicine Hospital of Zaghouan Zaghouan Tunisia; ^4^ Department of Radiology Mohammed Kassab National Institute of Orthopaedics Mannouba Tunisia

**Keywords:** blood hypertransfusion, extramedullary hematopoiesis, MRI, radiation therapy, spinal cord compression syndrome, thalassemia major

## Abstract

Extramedullary hematopoiesis is a normal compensatory reaction that can affect the epidural space, leading to spinal cord compression syndrome. MRI is the imaging method of choice for diagnosis and monitoring. Treatment is still controversial.

## Graphical abstract

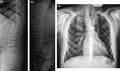

## 1 INTRODUCTION

Thalassemia is an autosomal recessive inherited condition that causes chronic anemia. It is common in the Mediterranean basin.^1^ Extramedullary hematopoiesis (EMH) is a common compensatory reaction to chronic hemolytic anemia that has been reported in many hematologic disorders such as hemolytic anemia especially thalassemia, myelofibrosis or myelodysplastic syndromes, leukemia, lymphoma, or after irradiation of bone marrow.^2^ Extramedullary hematopoiesis is more prevalent in men (5:1 male/female ratio) and usually involves the liver, spleen, and lymph nodes.^1^, ^2^, ^3^ Spinal cord compression (SCC) due to EMH is an extremely rare condition that can be life‐threatening and leads to permanent disability if left untreated. The management of SCC due to EMH remains controversial and includes blood transfusions, decompressive surgery, and radiotherapy.^1^ We report the case of a 20‐year‐old man with beta‐thalassemia major presenting with SCC due to EMH successfully treated by radiotherapy and blood transfusion.

## 2 CASE REPORT

A 20‐year‐old male with known thalassemia major, first diagnosed at the age of 6 months. He had a splenectomy in February 1997, after which his blood transfusion needs declined. Due to the asymptomatic nature of his condition, he escaped regular medical monitoring with poor compliance with his treatment.

For the past 6 months, he has had mechanical low back pain that has been progressively worsening for the last month, becoming inflammatory with prolonged morning stiffness. Moreover, he reported the appearance of bilateral low limb paresthesia and weakness. Otherwise, he had no history of trauma, no fever or weight loss, no upper limbs symptoms, and no bladder or bowl disorders.

Spine examination was difficult because of the pain. The visual analog scale (VAS) for pain was 9/10. Neurological examination revealed hypoesthesia of the lower limbs. Muscular strength at the lower limbs was rated at 3/5. The tone was normal. Tendon reflexes were present and symmetrical, and Babinski's reflex was negative. Anal sensitivity and contraction were normal.

Laboratory tests showed hemoglobin 5 g/dL, white blood cell count of 20 600 elements/mm^3^, and platelets 558 000 elements/mm^3^. The blood smear revealed anisopoikilocytosis, hypochromia, and nucleated red blood cells. Biochemical investigations showed biological inflammatory syndrome (ESR = 55 s, CRP = 5 mg/L). The rest of the biological tests were normal, and infectious investigation was negative.

**FIGURE 1:**
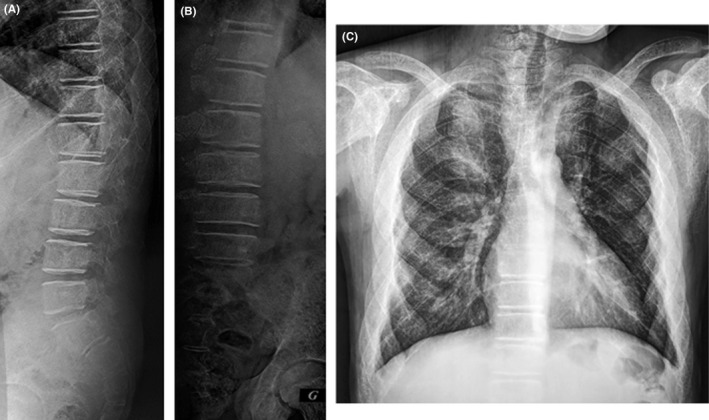
Spinal (A and B) and Chest ^©^ X‐ray: diffusely osteopenic skeletal structures, with expansion of the medullary spaces and a lace‐like trabecular pattern

**FIGURE 2:**
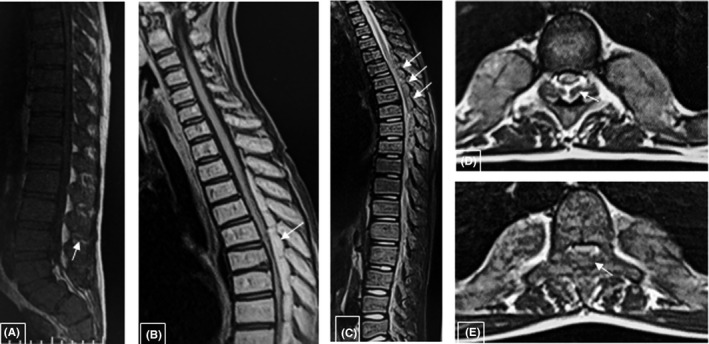
MRI sagittal T1 WI (A and B), sagittal T2WI ^©^, axial T2 WI (D and E) shows diffusely hypointense marrow (A), paraspinal and epidural masses extending from T2 to L2 causing cord compression regarding T5 and T6 (arrows), and an anterior epidural mass regarding S1 causing the compression of the filum terminale (2A arrow)

The plain X‐ray of the spine showed that skeletal structures were diffusely osteopenic, with expansion of the medullary spaces and a lace‐like trabecular pattern. (Figure 1) Magnetic resonance imaging (MRI) of the thoracolumbar spine revealed diffusely hypointense marrow(Figure 2A), paraspinal, and epidural masses extending from T2 to L2 causing cord compression regarding T5 and T6 (Figure 2B‐E), and an anterior epidural mass regarding S1 causing the compression of the filum terminale. (Figure 2A).

A diagnosis of thalassemia with EMH was established based on the MRI findings.

The patient was started on blood hypertransfusion. He received two unit of packed RBC/week. Surgical decompression was avoided due to the extent of the lesion and the neurological and hemodynamic risks. He was scheduled for radiation of the affected region of the spine and received a cumulative dose of 3000 cGy in six fractions (500 cGy per fraction).

**FIGURE 3:**
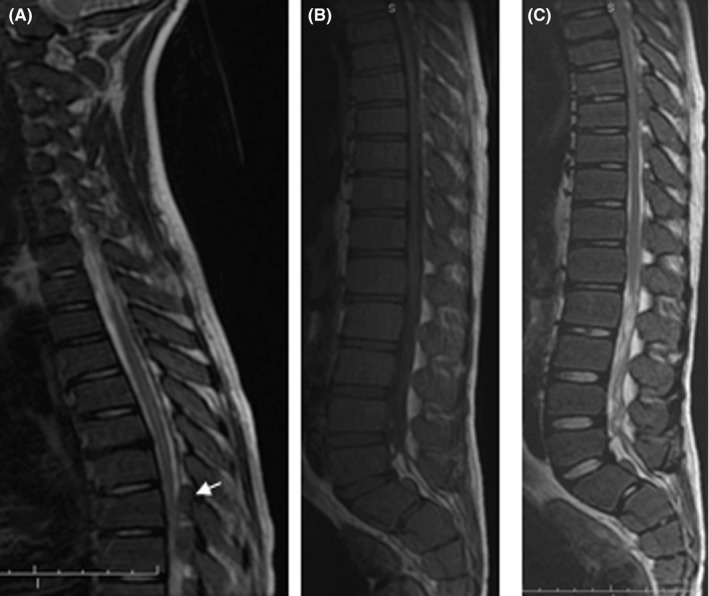
MRI sagittal T2 and T1 WI demonstrated significant improvement of the epidural masse

The follow‐up at 5 months showed a significant decline of the pain (VAS = 20%). The patient's hemoglobin was elevated from 5 g/dL to 9 g/dL and with no transfusion reaction having been noted. MRI demonstrated significant improvement of the epidural masses with no evidence of cord compression. (Figure 3).

## 3 DISCUSSION

Beta‐thalassemia is an inherited condition characterized by a limited synthesis of the β chains of hemoglobin.^4^ Extramedullary hematopoiesis is a common complication of thalassemia intermediate and under transfused patients in thalassemia major. It is the consequence of ineffective erythropoiesis.^5^ Extramedullary hematopoiesis can occur in any tissue of mesenchymal origin mostly in the liver, spleen, and lymph nodes and exceptionally in the adrenal glands, kidneys, dura mater, adipose tissue, and skin.^2^

The first documented case of EMH affecting the epidural region was reported in 1954 by Gatto et al^6^ This condition mainly affects the lower thoracic spine where the limited mobility and the narrow spinal canal predispose to SCC.^7^ Usually, the clinical presentation includes vertebral pain associated with radiculalgia. Gradually, neurological disorders such as paresthesia, motor impairment of the lower limbs, and even cauda equina syndrome with bladder and bowel disorders appear.^1^

Differential diagnoses include neurogenic tumors, primary tumors, metastases, and epidural abscesses.^8^ Although the definitive diagnosis remains histological, biopsy is reserved for subjects with severe SCC scheduled for laminectomy or in cases of doubtful diagnosis.^2^, ^3^, ^4^, ^5^, ^6^, ^7^, ^8^, ^9^ MRI is currently the imaging of choice for diagnosis and monitoring, exceeding the spinal CT scan. It shows a highly vascularized marrow lesion with an intermediate signal on the T1‐ and T2‐weighted sequences. In the case of older lesions, the signal strength becomes high due to fat infiltration.^2^

The management of this condition remains controversial due to its rarity. It includes decompressive surgery, radiation, blood transfusions, and hydroxyurea.^8^, ^9^, ^10^

Recurrent blood transfusions or hypertransfusion can reduce the need for extramedullary hematopoiesis and are thus used as a diagnostic tool.^8^, ^9^ However, they are often used in combination with other methods due to the often temporary and partial improvement.^7^, ^8^

Several cases reported in the literature have proved the efficiency of radiotherapy with significant clinical and radiological improvement.^9^ Modest doses of 1000‐3000 cGy are usually effective.^4^ However, radiation can lead to an initial worsening secondary to edema, which can be prevented by the addition of corticosteroids. It can also lead to a decrease in blood cell turnover, which must be controlled.^7^, ^8^ Moreover, a recurrence of EMH after radiation is reported in 19%‐37% of cases.^10^

Decompressive surgery may be used occasionally in severe life‐threatening cases that do not respond to other treatment. However, we must keep in mind the associated risks such as the hemorrhagic, neurological, and infectious risk as well as the risk of recurrence.^5^, ^6^, ^7^, ^8^, ^9^

Hydroxyurea, an inhibitor of the ribonucleotide reductase enzyme, can also be used. It acts by stimulating the synthesis of fetal hemoglobin.^5^, ^6^, ^7^, ^8^

## 4 CONCLUSION

Spinal cord compression resulting from EMH in patients with thalassemia major is a rare condition. It must be recognized early on the basis of clinical presentation and the results of the MRI to avoid irreversible neurological sequelae. The optimal management of EMH remains controversial. We strongly advocate low doses of radiotherapy as the treatment of choice for SCC secondary to EMH in combination with blood transfusion. Thus, surgery should be reserved for severe cases with acute neurological deterioration or in case of doubtful diagnosis despite adequate treatment.

## CONFLICT OF INTEREST

None declared.

## AUTHOR CONTRIBUTIONS

HF, KM, DK and WH: analyzed and interpreted the patient data and provided advice for treatment. HR: interpreted the first and the second MRI. HF: ensured the clinical follow‐up of the patient. LBA and HF: were major contributor in writing the manuscript; and all authors: read, revised, and approved the final manuscript.
